# Milestones in plant sulfur research on sulfur-induced-resistance (SIR) in Europe

**DOI:** 10.3389/fpls.2014.00779

**Published:** 2015-01-15

**Authors:** Elke Bloem, Silvia Haneklaus, Ewald Schnug

**Affiliations:** Federal Research Centre for Cultivated Plants, Julius Kühn-Institute, Institute for Crop and Soil ScienceBraunschweig, Germany

**Keywords:** nutrient induced resistance, S fertilization, plant S metabolism, fungal diseases, biotrophic and necrotrophic pathogens

## Abstract

Until the 1970's of the last century sulfur (S) was mainly regarded as a pollutant being the main contributor of acid rain, causing forest dieback in central Europe. When Clean Air Acts came into force at the start of the 1980's SO_2_ contaminations in the air were consequently reduced within the next years. S changed from an unwanted pollutant into a lacking plant nutrient in agriculture since agricultural fields were no longer “fertilized” indirectly by industrial pollution. S deficiency was first noticed in *Brassica* crops that display an especially high S demand because of its content of S-containing secondary metabolites, the glucosinolates. In Scotland, where S depositions decreased even faster than in continental Europe, an increasing disease incidence with *Pyrenopeziza brassicae* was observed in oilseed rape in the beginning 1990's and the concept of sulfur-induced-resistance (SIR) was developed after a relationship between the S status and the disease incidence was uncovered. Since then a lot of research was carried out to unravel the background of SIR in the metabolism of agricultural crops and to identify metabolites, enzymes and reactions, which are potentially activated by the S metabolism to combat fungal pathogens. The S status of the crop is affecting many different plant features such as color and scent of flowers, pigments in leaves, metabolite concentrations and the release of gaseous S compounds which are directly influencing the desirability of a crop for a variety of different organisms from microorganisms, over insects and slugs to the point of grazing animals. The present paper is an attempt to sum up the knowledge about the effect of the S nutritional status of agricultural crops on parameters that are directly related to their health status and by this to SIR. Milestones in SIR research are compiled, open questions are addressed and future projections were developed.

## Nutrient induced resistance

Already Justus von Liebig identified in 1873 the nutritional status of a crop as crucial for its susceptibility against diseases. Interactions between mineral elements and plant diseases are established for several macro- and microelements. An overview of current knowledge on the effect of mineral nutrition on plant diseases was compiled by Datnoff et al. (2007).

A sufficient nutrient supply is the first agricultural measure against infection and determines the course of pathogenesis. In general, the greatest benefit can be expected when all essential nutrients are applied in sufficient amounts; however, the response to a particular nutrient may be different when going from deficiency to sufficiency than from sufficiency to excess (Huber and Haneklaus, 2007). For nitrogen it was shown that fertilizer application above recommended rates can lead to significantly greater disease incidences (Walters and Bingham, 2007). Strengthening the natural plant resistance is an important aspect of fertilization practice and modern fertilizers deliver the possibility to individually treat each kind of nutrient deficiency by tailored-made products. All essential plant nutrients have a direct impact on plants, pathogens, and microbial growth so that all of them as well as their proportions are important in disease control and will affect disease incidence or severity (Huber and Haneklaus, 2007). This illustrates an important problem in investigating the metabolic background of sulfur induced resistance (SIR): Plant pathogen response is determined by several interacting factors—different nutrients and their interactions, soil parameters, climatic conditions, pathogens, water supply and much more. Therefore, it is nearly impossible to investigate the response to a certain pathogen in relation to S under natural conditions without having interacting parameters.

## Progress in research on sulfur induced resistance (SIR)

The fungicidal effect of foliar-applied elemental S (S^0^) was already discovered by William Forsyth in 1802 and S^0^ was used as the most important fungicide until the development of organic fungicides. The effects of foliar-applied elemental S have to be clearly distinguished from the health promoting effects of soil applied S on which SIR is based. The term SIR which denotes the reinforcement of the natural resistance of plants against fungal pathogens through triggering the stimulation of metabolic processes involving S by targeted soil-applied fertilizer strategies was first introduced by Schnug et al. (1995). In subsequent studies the term sulfur enhanced defense (SED) was used as synonym to prevent misinterpretation of the term resistance in a phytopathological context (Rausch and Wachter, 2005; Kruse et al., 2007).

Different research areas are of major relevance when investigating the background of SIR. The most important milestones in plant S research with respect to SIR are summarized in Table 1. Here important discoveries such as the detection of the *Foyer-Halliwell-Asada* pathway or the mustard oil bomb are listed as well as important technical developments.

**Table 1:** **Discoveries and progress in plant sulfur (S) research with respect to sulfur induced resistance (SIR) during the twentieth century**.

**Year**	**Scientific discoveries**	**References**
1802	• William Forsyth discovered the fungicidal effect of elemental S	Forsyth, 1802
1860	• S was recognized as an essential plant nutrient, required for growth	Woodard, 1922
1872	• Robert Angus Smith coined the term “acid rain”	Seinfeld and Pandis, 1998
1956	• The common structure of glucosinolates was discovered	Ettlinger and Lundeen, 1956
1973	• Elucidation of the major steps in glucosinolate biosynthesis	Underhill et al., 1973
1976	• First description of the *Foyer-Halliwell-Asada*-cycle	Foyer and Halliwell, 1976
1977	• *Agrobacterium tumefaciens*- mediated gene transfer	Chilton et al., 1977
1979	• SO_2_ exposure increase the glutathione content in sensitive trees	Grill et al., 1979
1982	• Description of the glutathione metabolism in higher plants and its function in transport, storage and detoxification of xenobiotics	Rennenberg, 1982
	• Detection of hydrogen sulfide (H_2_S) emissions from leaf tissue in response to L-cysteine feeding	Sekiya et al., 1982
1984	• Description of the stimulating effect of abiotic stress and the restrictive impact of S deficiency on synthesis of S containing secondary plant metabolites	Gershenzon, 1984
	• Description of the “mustard oil bomb,” a model of the subcellular organization of the glucosinolate-myrosinase system	Lüthy and Matile, 1984
1986	• Demonstration that leaf glucosinolates of *Brassica napus* can control fungal infection by *Leptospheria maculans*	Mithen et al., 1986
1989	• Plants can take up and use atmospheric H_2_S as S source	De Kok et al., 1989
1990	• Localization of the γ-glutamylcysteine synthetase in higher plants	Hell and Bergmann, 1990
1994	• The term “sulfur induced resistance” (SIR) was introduced after field trials unraveled a relationship between S nutrition and plants susceptibility toward fungal diseases	Schnug et al., 1995
	• Significance of glutathione in plants under stress was demonstrated	Rennenberg and Brunold, 1994
	• Concept of “biofumigation” was developed	Angus et al., 1994
1995	• Isolation of three sulfate transporters for sulfate uptake by plant roots	Smith et al., 1995
1996	• Detection and cellular localization of elemental S in disease resistant genotypes of *Theobroma cacao*	Cooper et al., 1996
1999	• Overexpression of serineacetyltransferase (SAT) caused increased cysteine and glutathione contents accompanied by an increased resistance to oxidative stress	Blaszczyk et al., 1999
2000	• Interaction of sulfate reduction with N nutrition and major role of *O*-acetylserine in this regulation was shown at the transcriptional level	Koprivova et al., 2000
2001	• Identification and biochemical characterization of *Arabidopsis thaliana* sulfite oxidase	Eilers et al., 2001
2003	• Application of DNA macroarray technique to investigate the gene-to-metabolite networks regulating the S metabolism of *Arabidopsis*	Hirai et al., 2003
2004	• The regulatory function of the *O*-acetylserine(thiol)lyase (OAS-TL) in the S assimilation pathway was shown	Wirtz et al., 2004
2005	• Introduction of the term “sulfur enhanced defense” (SED)	Rausch and Wachter, 2005
	• Higher susceptibility of S deficient oilseed rape for different pathogens	Dubuis et al., 2005
	• The link between S assimilation and the stress hormone jasmonate (JA) was proven by macroarray technique	Jost et al., 2005
2006	• Identification of PAD2 as a γ-glutamylcysteine synthetase and the importance of glutathione in pathogen defense	Parisy et al., 2006
2009	• Indole glucosinolate biosynthesis and hydrolysis is required for callose accumulation in response to microbial pathogens	Clay et al., 2009
2012	• A shift from plant COS uptake to COS release with fungal infection	Bloem et al., 2012
	• Regulatory role of cytosolic cysteine/cytosolic OAS-TL in plant immune response	Alvarez et al., 2012; Tahir et al., 2013

The achievements made in gene transfer, by which the possibility to work with genetically modified plants was established, as well as the elucidation of the *Arabidopsis* genome promoted the progress in plant S research tremendously (Chilton et al., 1977). Experimentation with knock-out mutants delivered deep insight into plant metabolism and cross-talk between different pathways (Thomma et al., 1998; Kopriva, 2006; Parisy et al., 2006).

A lot of efforts were undertaken to understand the S assimilation pathway in plants, the transport of S into plants, and the storage and regulation of the S metabolism (Table 1). Since the completion of the *Arabidopsis* genome research has made considerable progress.

For example a range of S transporters carrying S containing metabolites within and between cells and over long-distance have been characterized, some of them just recently (Gigolashvili and Kopriva, 2014). In glucosinolate research the biosynthesis as well as its regulation was nearly explained in the last years (Halkier and Gershenzon, 2006).

Technical progress such as the development of macroarray hybridization can be seen as a further important milestone. Jost et al. (2005) recorded the reaction of more than 2000 selected genes of *Arabidopsis thaliana* to methyl jasmonate (JA) elicitation, a signaling compound in host-pathogen interactions. The authors could show that S-related genes were even more up-regulated due to methyl JA treatment than stress-related genes and that more than one pathway is involved in plant stress response. Gene expression of the ascorbate and glutathione metabolic pathways increased in response to JA as well as the synthesis of indole glucosinolates (Sasaki-Sekimoto et al., 2005). Moreover it was shown that imbalances in cytosolic cysteine alter the expression of groups of genes involved in pathogen response (Alvarez et al., 2012). Therefore, macroarray analysis delivers the opportunity to investigate and understand the network and cross-talk of metabolic pathways.

But despite of these great advances in scientific discoveries and technologies delivering several pieces of the puzzle of SIR, many questions remain open. It is still under discussion which reactions or compounds are responsible for the higher resistance of plants in relation to the S supply and how it is possible to induce a higher resistance and use this by advanced fertilizer application.

## Physiological background of SIR

Plants have developed several defense mechanisms in response to stress and react to a certain pathogen attack through a combination of constitutive and inducible defense with S-containing compounds being involved compiled by Bloem et al. (2005). In principle plants have three major strategies to combat pathogens: cell wall strengthening, apoplastic defense for inhibition of microbial enzymes and poisoning of the pathogen by toxic compounds like phytoalexins (Huckelhoven, 2007).

Initial pathogen recognition causes responses such as oxidative burst with the production of reactive oxygen species (ROS) and cell wall lignification (Swarupa et al., 2014). ROS serve as major signaling molecules in plant defense and are closely linked to the S metabolism via the *Foyer-Halliwell*-*Asada* pathway where glutathione is involved in the detoxification of ROS (Foyer and Halliwell, 1976). Via this link to ROS the S metabolism is linked to pathogen recognition and activation of the defense network.

The complexity of plant stress responses became obvious in several infection trials. S metabolites such as cysteine, glutathione, gaseous S emissions, phytoalexins, glucosinolates, and elemental S depositions have been investigated for their role in plant defense and how targeted S applications may prompt and enhance crop resistance to fungal pathogens (Bloem et al., 2007; Haneklaus et al., 2007, 2009). For most S containing metabolites a direct antifungal mode of action was proven (Table 2). Cysteine is the main precursor for all S containing compounds and is directly linked to stress response via its function related to systemic acquired resistance (Luckner, 1990). Cysteine displays a regulatory function in pathogen defense. It was shown that a specific cytosolic cysteine content is mandatory for the initiation of the plant immune response to pathogens and a link to the hypersensitive response (HR) was proven (Alvarez et al., 2012).

**Table 2:** **Possible mode of action of S-containing plant compounds in stress resistance and in response to fungal infection**.

**Compound**	**Mode of action in stress resistance and after fungal infection**	**References**
Cysteine	Precursor for all relevant S containing metabolites	Luckner, 1990; Bloem et al., 2007; Alvarez et al., 2012
	−Cytosolic cysteine has a regulatory function in the establishment and signaling of the plant response to pathogens	
	−Increase with fungal infection	
	−Link to salicylic acid and by this to systemic acquired resistance via CoASH and essential for the initiation of the hypersensitive response (HR)	
Glutathione	Participation in antioxidative defense	Edwards et al., 1991; Rea et al., 1998;Leustek and Saito, 1999; Cobbett, 2000; Foyer and Rennenberg, 2000; Vanacker et al., 2000
	−Detoxification of xenobiotics by targeting them into the vacuole	
	−Involved in phytochelatine biosynthesis/ detoxification of heavy metals	
	−Messenger in the hypersensitive response (HR)	
	−Rapid accumulation after fungal attack	
S-containing volatiles	H_2_S causes disturbances in redox reactions	Bloem et al., 2007, 2012
	−Release of H_2_S and COS increased with fungal infections	
S-rich proteins	Pathogen-induced or constitutive expression (defensins)	Hughes et al., 2000; Stec et al., 2004; Kruse et al., 2007
	−Thionins are enhanced locally and systemically after infection	
	−Toxic mode of thionins: disruption of the cell wall structure; generation of ion channels	
Phytoalexins	*De-novo* synthesis after pathogen attack	Kuć, 1994
S^0^	S^0^ accumulates after fungal infection in vascular tissue	Beffa, 1993; Cooper et al., 1996; Williams et al., 2002
	−Disturbances of the respiratory chain	
	−Oxidation of sulfhydryl groups	
Glucosinolates	Their degradation products (isothiocyanates) exhibit a toxic and repellent effect → reason for its use in biofumigation	Mithen, 1992; Wallsgrove et al., 1999

Glutathione displays a central function in plant defense as well: it is an important redox buffer in cells as it exists in a reduced form (GSH) which can react with another molecule of reduced glutathione (GSH) to form the oxidized disulfide form (GSSG) and which is restored by the enzyme glutathione reductase (Leustek et al., 2000). The ratio of reduced to oxidized glutathione delivers already an important information as it decreases under stress conditions that consume reducing equivalents. Moreover, glutathione is supposed to be involved in stress signaling, the detoxification of xenobiotics, it is the precursor of phytochelatines, which are important for heavy metal detoxification, acts as transport and storage form of reduced S and has a regulatory function in S assimilation (Leustek et al., 2000). These manifold functions illustrate the major importance of glutathione in plant S metabolism and stress response.

A direct antifungal mode of action was determined for S-rich proteins, phytoalexins such as camalexin, elemental S and the degradation products build from glucosinolates (Mithen, 1992; Kuć, 1994; Cooper et al., 1996; Wallsgrove et al., 1999; Hughes et al., 2000; Williams et al., 2002; Stec et al., 2004; Glawischnig, 2007). The toxicity of S-rich proteins such as thionins is explained by their ability to generate ion channels in cell membranes of pathogens and by this disturbing ion concentration gradients and cellular homeostasis (Shai, 1999; Hughes et al., 2000).

The antifungal mode of action of S^0^ can be explained by its lipophilic character. S^0^ may enter directly through the fungal cell wall disturbing redox reactions in the metabolism of the pathogen (Beffa, 1993). Beffa (1993) suggested that the fungicidal action of S^0^ is mainly related to the oxidation of important sulfhydryl groups. S^0^ depositions in the vascular tissue of resistant varieties of *Theobroma cacao* in response to infection by *Verticillium dahliae* were considered as defense reaction causing the resistance of these varieties (Cooper et al., 1996; Resende et al., 1996).

Native glucosinolates display no fungal toxicity in contrast to their hydrolysis products, the isothiocyanates (ITC), which display a strong antimicrobial activity (Manici et al., 1997). Fungal inhibition is caused by irreversible reactions of ITC's with functional groups of proteins resulting in enzyme inactivation (Brown and Morra, 1997). In accordance with this not only the biosynthesis of indole glucosinolates was up-regulated by ethylene signaling after pathogen recognition in *Arabidopsis* but also the expression of myrosinase enzymes which catalyze their hydrolysis (Clay et al., 2009). Additionally the biosynthesis of indole glucosinolates was shown to be required for callose depositions in response to microbial pathogens (Clay et al., 2009). Therefore, glucosinolate biosynthesis seems to be involved in pathogen defense in more than one way in glucosinolate containing plants.

The concentrations of all S containing metabolites, the amino acids cysteine and methionine as well as primary and secondary S compounds were reduced with S deficiency or can be increased by S fertilization (Salac et al., 2005; Bloem et al., 2007). It was observed that the gas exchange of H_2_S and carbonyl sulfide (COS) between plants and atmosphere changed in relation to S supply and fungal infection. As long as enough S is available plants release H_2_S into the atmosphere. This happens most likely to reduce excess S in their metabolism or as a signal molecule (Rennenberg, 1984; Bloem et al., 2012). Under conditions of S deficiency plants take up and use gaseous S compounds from the ambient air (De Kok et al., 1989).

**Figure 1:**
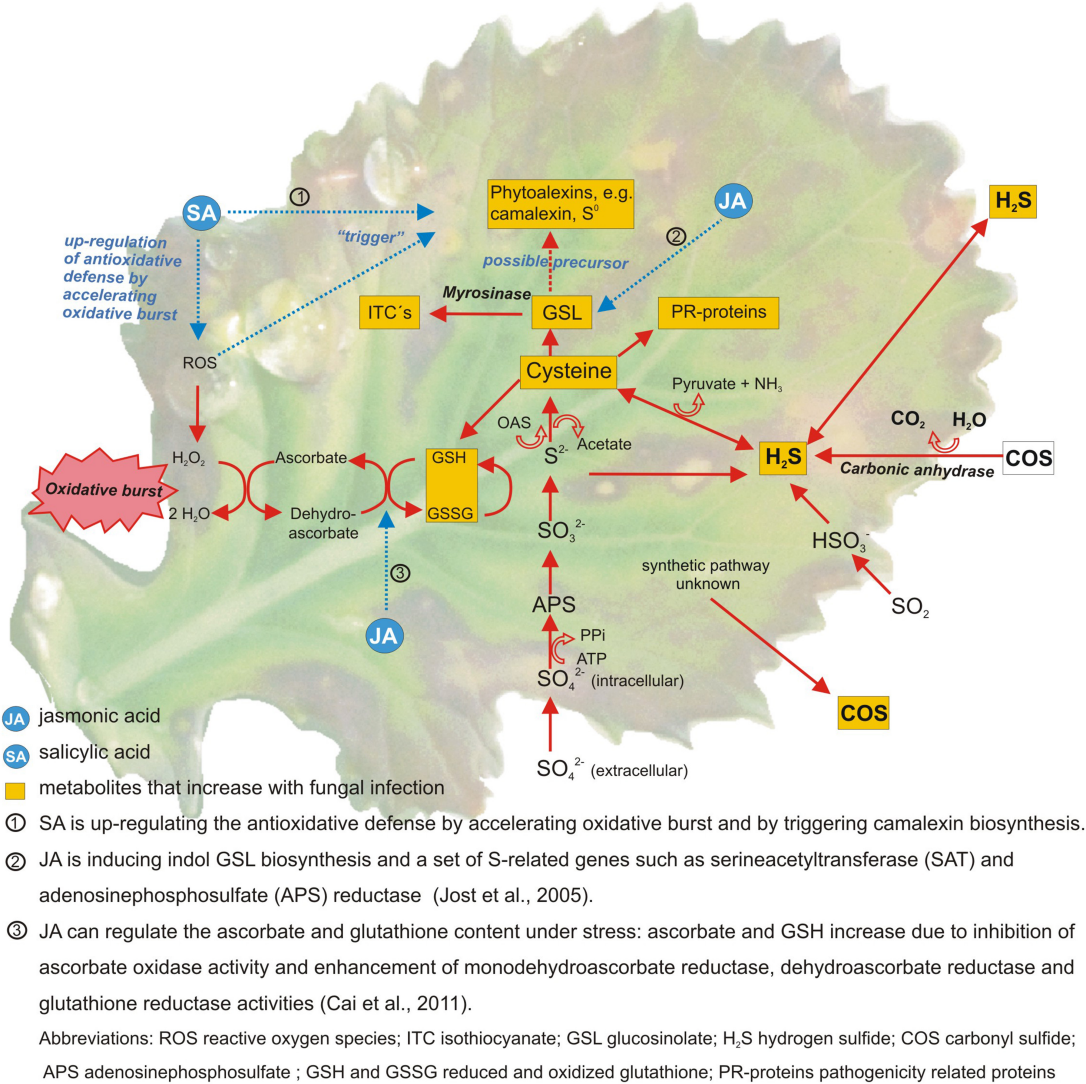
**Sulfur metabolites and pathways involved in the defense against fungal pathogens in *Brassica* species**. Metabolites in yellow boxes were found to increase after fungal attack (Williams and Cooper, 2004; Glazebrook, 2005; Jost et al., 2005; Kruse et al., 2007; Bloem et al., 2012).

Linear relationships were determined between the S supply and most of the mentioned S containing compounds. When next to the S supply a fungal infection was studied the results became less conclusive (Bloem et al., 2004; Salac et al., 2005). In many trials S fertilization decreased fungal infection (Wang et al., 2003). But in some trials no effect on disease severity could be determined despite of the fact that a stress response occurred, indicated by changes in the S metabolism (Salac et al., 2005). The S metabolism is only one branch of the overall plant stress response. Several other pathways and metabolites are involved (Bennett and Wallsgrove, 1994; Morrissey and Osbourn, 1999). Amongst others flavonoids and phenolics are shown to be major biochemical marker against fungal infections (Shanmugam et al., 2010; Datta and Lal, 2012). Cell wall strengthening is another important resistance response against fungi as it helps to inhibit pathogen entry. Accumulation of cell wall-bound phenolics, the monomers of lignin, is part of this process (Swarupa et al., 2014). It was shown that cell wall-bound phenolics increase together with soluble phenolics in plant tissue after fungal infection (Huckelhoven, 2007). Moreover several studies show a close link between the S metabolism, mineral deficiency or increased internal demand and hormonal signaling by methyl jasmonate and possibly other hormones (Hirai et al., 2003; Saito, 2004; Jost et al., 2005) (Figure 1).

Table 3 gives an example which changes occur in the S metabolism in response to S fertilization and fungal infection (Bloem et al., 2012). *Brassica napus* was artificially infected by *Sclerotinia sclerotiorum* and the plants displayed strong symptoms of S deficiency without S application.

**Table 3:** **Impact of S nutrition and fungal infection with *Sclerotinia sclerotiorum* on the S status, S-containing metabolites and the release of gaseous S compounds from *Brassica napus* (variety *Heros*) at stem elongation (data derived from Bloem et al., 2012)**.

		**Total S**	**SO_4_-S**	**Cysteine**	**γ-GC**	**GSH^1^_tot_**	**H_2_S^2^**	**COS^2^**
		**[mg g^−1^ dw]**		**[nmol g^−1^ dw]**			**[pg min^−1^ g^−1^ dw]**	
S fertilization	0	0.74 b	0.11 b	37.4 b	87.6 a	276 b	−91 b	−63 a
[mg pot^−1^]	250	5.63 a	1.34 a	232.0 a	39.8 b	2370 a	41 a	−174 a
Infection with	no	4.28 a	0.73 a	236.0 a	88.4 a	1383 b	41 a	−174 b
*Sclerotinia sclerotiorum*	yes	2.75 b	0.83 a	114.2 b	38.9 b	1851 a	123 b	382 a

1 *GSH_tot_, total glutathione content*.

2 *The gas measurement was performed on non-infected control plants to determine the effect of S fertilization and from S fertilized plants that were infected for 2 days for the impact of infection. Sulfur contents and metabolites were determined in leaf material of B. napus while the gas release was measured from whole intact plants*.

With increasing S supply total S and SO_4_-S increased in leaves as well as the cysteine and glutathione content (Table 3). Only γ–glutamylcysteine, which is an intermediate in the biosynthesis of glutathione, was lower with S fertilization indicating to a fast turn-over to build glutathione under these conditions. H_2_S exchange shifted from uptake, indicated by the negative value in S deficient plants, to H_2_S release in the fertilized ones. COS was taken up in S fertilized as well as in non-fertilized plants.

Infection with *S. sclerotiorum* caused significant changes in the S metabolism. The total S content decreased as well as cysteine and γ–glutamylcysteine while glutathione significantly increased. Additionally plants were analyzed for their potential to take up or release H_2_S and COS in the first days after infection (Bloem et al., 2012). In Table 3 the data from 2 days after infection are shown when the strongest plant response was observed. H_2_S release was significantly increased by infection. The change in COS was even more striking as COS was changed from uptake to release (Bloem et al., 2012). The data clearly revealed that plants responded to the infection by several changes in their S metabolism. Nevertheless, the visual scoring revealed that the infection rate was not reduced by the higher S supply at this stage of infection (see Bloem et al., 2012).

Likewise Raj and Srivastava (1977) showed that the total S content of infected tissue of *Brassica juncea* was inversely correlated with the pathogenicity of different isolates of *Macrophomina phaseolina* and suggested that the pathogens are able to metabolize S from the host plant. Losses in total S with fungal infection could be also explained by the release of gaseous S compounds like shown in Table 3. In some studies also a higher total S content was observed in response to infection indicating to an up-regulation of the S assimilation due to infection. Most likely it is the timing of sampling or the degree of infection which determine if an increase or decrease of a compound is determined in response to infection as a cascade of reactions takes place (Bloem et al., 2007).

A direct relationship between fungal infection and S metabolism as shown exemplary in Figure 1 was also found for other host-pathogen interactions. Infection of oilseed rape with *Pyrenopeziza brassicae* increased the cysteine and glutathione content in leaves as well as the activity of the L-cysteine-desulfhydrase, an enzyme that releases H_2_S during cysteine degradation (Bloem et al., 2004). A higher release of H_2_S after fungal infection was determined in grapes (*Vitis vinifera* L.) infected by *Uncinula necator* (Bloem et al., 2007). Gaseous S compounds seem to be involved in stress response but to date their function is not fully understood. A possible role could be in stress signaling or as regulatory compounds comparable to the effect in mammalian cells where H_2_S is involved in the regulation of the intracellular redox-homeostasis and glutathione generation (Ju et al., 2013).

Also Kruse et al. (2007) determined a steep and fast increase not only for H_2_S, but also for cysteine, glutathione and phytoalexins during the initial phase of pathogenesis. The important role of cysteine in pathogenesis was proven by Alvarez et al. (2012) and Tahir et al. (2013). Alvarez et al. (2012) could show that mutants with increased cytosolic cysteine content are resistant to biotrophic as well as necrotrophic pathogens, while mutants with decreased cytosolic cysteine contents are more susceptible. Also Tahir et al. (2013) found that decreased cytosolic cysteine contents resulted in enhanced disease susceptibility against infection with virulent and non-virulent *Pseudomonas syringae* strains.

Though the sequence, magnitude and efficacy of all individual S metabolites involved in the activation and strengthening of plant defenses by S fertilization are not yet fully known, these could be released in a chain reaction triggered by the pathogen and mediated by the S status of the plant (Haneklaus et al., 2006). It seems possible that infection triggers the activation of all effective resistance mechanisms of the host.

**Figure 2:**
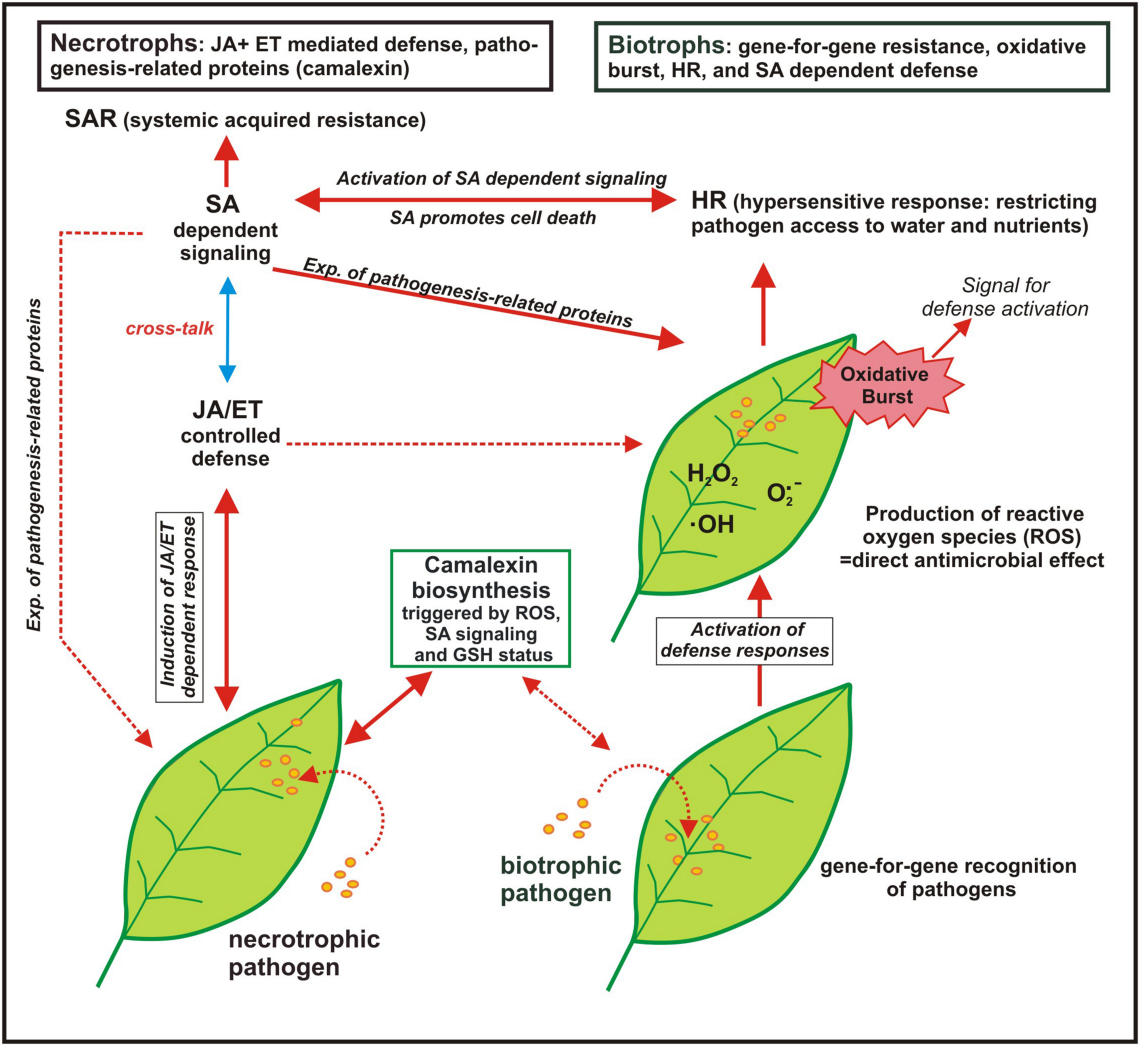
**Model of the response of plants to biotrophic and necrotrophic plant pathogens (adapted from Glazebrook (2005): displayed are the interactions of *Arabidopsis* with the biotrophs *Peronospora parasitica* and *Erysiphe* ssp. and with the necrotrophs *Alternaria brassicicola* and *Botrytis cinerea*; SA, salicylic acid, JA, jasmonic acid, ET, ethylene; broken line arrows indicate to a possible interaction but which was not found in the chosen experiments while the solid line arrows indicate to the observed plant-pathogen-response)**. The defense reaction of *Arabidopsis* against biotrophic pathogens start with gene-for-gene recognition of the pathogen followed by rapid activation of defense and the production of reactive oxygen species (ROS), the so-called “oxidative burst,” which is by self a signal for defense activation. ROS production is connected with the hypersensitive response (HR), also called “programmed cell death,” which limits the access of biotrophs that feed on living tissue to water and nutrients. HR is associated with the activation of the salicylic acid (SA) dependent signaling pathway that is connected with systemic acquired resistance (SAR) and the expression of pathogenesis-related proteins. For necrotrophic pathogens a different defense line takes place as they feed on dead plant tissue and host cell death is not predicted to limit their growth. Defense against necrotrophic pathogens is mainly mediated by JA and ET controlled defense as well as production of phytoalexins such as camalexin. The broken line arrows indicate that also mixed defense lines are possible for other biotrophic or nectrotrophic pathogens.

Trials where the effect of S nutrition on fungal infection was studied are compiled in Table 4. Plant pathogens are often divided into biotrophs and necrotrophs despite of the fact that there are several transitions. Biotrophs feed on living host tissue while necrotrophs cause die-off and feed on the remains (Glazebrook, 2005). SIR was proven for biotrophic and necrotrophic pathogens (Table 4). Different mechanisms in pathogen response are important for biotrophic and necrotrophic pathogens and a schematic model is summarized in Figure 2.

**Table 4:** **Influence of soil S application on pathogen development of different host pathogen interactions**.

**Host**	**Pathogen**	**Pathogen classification^1^**	**Lifestyle**	**Trial^2^**	**S-fertilization**	**Change in pathogen development**	**References**
**CONFIRMED SIR**							
*Brassica napus* L.	*Sclerotinia sclerotiorum*	A	necrotrophic (heterotrophic)	PT	120 mg S kg^−1^ soil	Disease index (DI) was reduced by 5% in comparison to a control without S application	Wang et al., 2003
*Zea mays*	*Bipolaris maydis* (Southern leaf blight)	A	necrotrophic	PT	120 mg S kg^−1^ soil	DI was reduced by 37% in comparison to a control without S application	Wang et al., 2003
*Brassica napus* L.	*Botrytis cinerea* (Gray mold)	A	necrotrophic	VWC	0.5 mM MgSO_4_	Lesions were 24-times larger in S-starved plants of cultivar *Express* and 3.7-fold larger in cultivar *Bienvenue*	Dubuis et al., 2005
*Brassica napus* L.	*Leptosphaeria maculans*	A	facultative necrotrophic, initially biotrophic	VWC	0.5 mM MgSO_4_	Lesions were 1.9-times larger in S-starved plants (cultivar *Bienvenue)*	Dubuis et al., 2005
*Arabidopsis thaliana*	*Alternaria brassicicola*	A	necrotrophic	WC	50 μM vs. 500 μM SO_4_	DNA from *A. brassicicola* was 3-time more abundant on plants grown on 50μM SO_4_ in comparison to plants grown on 500μM	Kruse et al., 2012
*Brassica napus* L.	*Pyrenopeziza brassicae*	A	hemi-biotrophic	FT	Plots with and without S fertilization	A non-resistant and a resistant oilseed rape variety were compared with and without S application and fungicide treatment: the non-resistant variety showed a much stronger response to fungicide under S deficiency	Schnug et al., 1995
*Solanum lycopersicum* L.	*Verticillium dahliae*	A	hemi-biotrophic	WC	0.016 mM vs. 25 mM K_2_SO_4_	Supra-optimal S supply significantly reduced the number of infected cells and the amount of *V. dahliae* gDNA in vascular tissue of the hypocotyl	Bollig et al., 2013
*Vitis vinifera* L.	*Uncinula necator*	A	obligate biotrophic	FT	250 or 500 kg S^0^ ha^−1^ (soil applied)	Proportion of infected leaves and berries decreased by more than 80% with soil S application	Haneklaus et al., 2007
*Solanum tuberosum* L.	*Rhizoctonia solani*	B	necrotrophic	FT	50 kg S^0^ ha^−1^ (soil applied)	Soil applied S^0^ reduced infection rate by 41% in comparison to control without S application	Klikocka et al., 2005
*Triticum aestivum* L.	*Rhizoctonia cerealis*	B	necrotrophic	PT	120 mg S kg^−1^ soil	DI was reduced by 44% in comparison to a control without S application	Wang et al., 2003
*Brassica napus* L.	*Peronospora parasitica*	O	obligate biotrophic	FT	100 kg S ha^−1^	Decrease in disease incidence and severity was found	Salac et al., 2005
*Brassica napus* L.	*Phytophthora brassicae*	O	hemi-biotrophic	VWC	0.5 mM MgSO_4_	Lesions were 3.3-times larger in S-starved plants of cultivar *Express*	Dubuis et al., 2005
**CONFLICTING RESULTS OF S FERTILIZATION ON PATHOGEN DEVELOPMENT**							
*Gossypium* L.	*Fusarium oxisporum* (Fusarium wilt)	A	necrotrophic	PT	160 mg S kg^−1^ soil	DI was reduced by 8% in comparison to a control; with all other S rates (40, 80, 120 mg S kg^−1^ soil) DI was higher than in the control	Wang et al., 2003
*Brassica napus* L.	*Leptosphaeria maculans*	A	facultative necrotrophic, initially biotrophic	FT	100 kg S ha^−1^	Depending on season, year and site controversial effects on disease incidence and severity were found	Salac et al., 2005
*Gossypium* L.	*Verticillium dahliae*	A	hemi-biotrophic	PT	40 mg S kg^−1^ soil	DI was reduced by 47% in comparison to a control, but with higher S application rates DI increased again and was significantly higher than in the control when 160 mg S kg^−1^ soil were applied	Wang et al., 2003
*Brassica napus* L.	*Pyrenopeziza brassicae*	A	hemi-biotrophic	FT	100 kg S ha^−1^	Depending on season, year and site controversial effects on disease incidence and severity were found	Salac et al., 2005

1 *Pathogen classification: A, Ascomycete; B, Basidiomycete; O, Oomycete*.

2 *Trial: PT, Pot trial; WC, Water culture; FT, Field trial; VWC, Vermiculite water culture*.

Generally defense reactions that cause the die-off of cells such as oxidative burst and hypersensitive response (HR) are only beneficial when repelling the attack of a biotrophic pathogen. In contrast, it is not predicted that the cell death of a host plant will limit the growth of necrotrophic pathogens. It is the opposite way round; necrotrophic fungi can elicit a defense response such as oxidative burst in a susceptible host plant causing necrosis (Winterberg et al., 2014). SA dependent defense is more frequently observed against biotrophs and JA/ET dependent defense against necrotrophs but there are exceptions. The fact that pathogenesis-related proteins are not expressed and JA dependent signaling is not activated against a special biotrophic pathogen does not mean that they are not active in case they are triggered (Glazebrook, 2005). S containing compounds are involved in both defense lines (see also Figure 1). Glutathione is involved in detoxification of ROS, many pathogenesis-related proteins contain S (phytoalexins, thionins, defensins) and SA needs coenzyme A (CoASH) as a precursor. Therefore, it is hardly possible to predict the efficacy of S against special fungi based on the lifestyle of the pathogen.

It is hard to explain why in some trials a clear relationship between the S supply and the extent of fungal infection was found whilst in others with the same pathosystem no such response was observed. Probably it is the timing and extent of plants defense response which decides over resistance or susceptibility while the nutritional status of the crop determines the extent of defense. Moreover, the type of pathogen and its pathogenicity, infection severity and other environmental factors are important as well.

## Practical relevance of SIR

Optimizing the S nutritional status of a plant is equivalent of enhancing the capability of a plant to cope with stress. The identification of the mechanisms causing SIR is an important milestone for a sustainable agricultural production as the input of fungicides can be minimized by crop specific S fertilization and a higher resistance due to S will not be rapidly broken by new pathotypes. It is possible to optimize the S nutrition without understanding all mechanisms underlying SIR. For winter crops a first S application in autumn was shown to be advantage with respect to disease resistance followed by the regular S application in spring. An increasing S supply is associated with higher contents of cysteine, glutathione, H_2_S, and glucosinolates in *Brassica* crops so that plants with a higher content of phytoanticipins might not only have *a priori* a better protection against pathogens, but also be able to activate resistance mechanisms more rapidly and intensely. In addition, an instantly high S supply satisfying the elevated S demand after a fungal attack may play a pivotal role in SIR, even when the nutrient demand of the crop is well exceeded by such an S application (Haneklaus et al., 2009).

## Open questions and future projections

It is generally difficult to assign a change in plant metabolism to a specific stress factor, as usually a variety of abiotic and biotic stress factors occur at the same time and can induce antagonistic responses resulting in accumulation, degradation and consumption of primary and secondary metabolites. Therefore, standardized experimental conditions are important to improve comparability of results.

Moreover, more field studies and infection trials are necessary accompanied by molecular research to unravel the relationship between S supply and fungal infection and by this to enable researchers and farmers to adopt the results into new fertilizer concepts. There are many unknowns affecting plant response such as timing of application, kind of fungal pathogen, crop species, climatic conditions, and cross-talk with other macro- and micronutrients. Therefore, to date it is not possible to induce a stress response by S application that will certainly reduce or prevent a crop from fungal infection. It is necessary to further elucidate the cross-talk between different pathways to understand which other parameters need to be optimized in order to reach the full potential of plants own pathogen defense.

Much more work in the field of phytopathology is necessary to solve the questions why only some pathogens are affected by S nutrition and which is the exact mode of action by which the S supply is affecting fungal pathogens. Could be the lifestyle (biotrophic, necrotrophic, heterotrophic) of a pathogen important for plants defense in relation to S or is the timing of infection in relation to plant development most important for the course of infection? Which part of metabolite changes is caused by the host and which one by the pathogen? These are only some of the manifold topics and questions where further research is necessary.

Moreover, it is not possible to transfer all results obtained from research with model plants such as *Arabidopsis* to other species so that studies on major agricultural crops are important especially as most agricultural crops do not contain glucosinolates.

Nevertheless, the manifold results, which point to a relationship between S nutrition and crop resistance, indicate that in future the crucial factors will be identified. There are still some agricultural diseases where the efficiency of chemical fungicides is limited. For example currently, no fungicides are available to control *Verticillium* wilt. Therefore, fertilizer strategies which improve the plants potential and resistance against fungal diseases are still of high importance not only in organic farming but in conventional agriculture as well.

### Conflict of interest statement

The authors declare that the research was conducted in the absence of any commercial or financial relationships that could be construed as a potential conflict of interest.
